# Telemedicine Awareness Among Chennai-Based Dentists: A Web-Based Questionnaire Survey

**DOI:** 10.7759/cureus.65349

**Published:** 2024-07-25

**Authors:** Vishveshwar B, Debayan Ghosh, Sathya Kumar M, Magesh K T, Indumathi K P, Aravindhan R, Sivachandran A, Mitthra S

**Affiliations:** 1 Oral Pathology and Microbiology, Sri Ramaswamy Memorial (SRM) Kattankulathur Dental College and Hospital, SRM Institute of Science and Technology (SRMIST), Chennai, IND; 2 Public Health Dentistry, Sri Ramaswamy Memorial (SRM) Kattankulathur Dental College and Hospital, SRM Institute of Science and Technology (SRMIST), Chennai, IND; 3 Conservative Dentistry and Endodontics, Sri Ramaswamy Memorial (SRM) Kattankulathur Dental College and Hospital, SRM Institute of Science and Technology (SRMIST), Chennai, IND

**Keywords:** covid-19, patient care, telemedicine, awareness, chennai-based dentists, teledentistry

## Abstract

Objectives: Teledentistry has emerged as a crucial tool to address oral health needs amidst social distancing measures and lockdowns. Teledentistry during COVID-19 underscores its potential as an adaptive solution, transforming the delivery of dental care and providing a platform for maintaining oral health while prioritizing public safety. The purpose of this study is to investigate the views of Chennai dental professionals on teledentistry, which may serve as a foundation for advancements in patient care and virtual dentistry in the future, and to evaluate awareness, usefulness, and data security regarding telemedicine among Chennai dental professionals using a pretested questionnaire.

Methods: By distributing a pretested web-based questionnaire to licensed dental professionals in Chennai, a descriptive cross-sectional study was conducted. The results were statistically analyzed and evaluated.

Results: A total of 90 dentists in Chennai responded to the questionnaire, out of which 65 (72.2%) were aware of teledentistry and 32 (35.6%) practiced teledentistry. Around 25 to 29 (30%-37%) dentists were very much concerned about obtaining patients’ consent, digital forgery, confidentiality, and hardware reliability in teledentistry.

Conclusion: This is the first kind of study ever done among dentists in Chennai, which shows enormous work is required to create awareness and knowledge among dental professionals. To inform dentists and the general public about teledentistry, targeted advertisements pertaining to the same are required.

## Introduction

The Greek word "tele" means "distance," and the Latin word "mederi" means "to heal." Telemedicine has been dubbed "healing by wire" by the Time magazine. Telemedicine was formerly viewed as "futuristic" and "experimental," but it is now a reality and here to stay. There are many uses of telemedicine in patient care, public health, education, research, and administration. Access to prompt, high-quality specialty medical care is a challenge for many people who live in rural and isolated places across the world. Because specialized physicians are more likely to be found in densely populated urban regions, residents of these places frequently have inadequate access to specialty healthcare [1,2]. Telemedicine has promise for bridging this gap and enabling healthcare in these isolated places [3].

One area of telemedicine is teledentistry. There are numerous subspecialties within teledentistry, including telepathology, teleradiology, teleorthodontia, telesomatology, and teleoral surgery. A new area of dentistry, teledentistry, employs communication networks and information-based technology to provide healthcare to the public. The appropriate digital communication channels are used to deliver fundamental dental care information. It may serve as a well-known platform for professional-to-professional contact and, in the long run, provide dentistry students with superior instructional resources. Additionally, it aids in educating patients about the fundamentals of dental care, enhancing the quality of the facilities for patient care [4,5].

The COVID-19 pandemic has catapulted telemedicine to the forefront of healthcare services as it allows for the provision of care when distance separates the provider and patient. This increased reliance on telemedicine has led to rapid promotion and changes in laws regarding its coverage. A key tactic for maintaining dental health during the COVID-19 outbreak has been teledentistry. Many nations, including China, US, Japan, Italy, and UK, have adopted it extensively. With several advantages for both patients and dentists, teledentistry uses electronic information and communication technology to deliver dental care remotely. It speeds up consultations and treatment planning, lowers the cost and anxiety associated with travel, and expands access to oral health care [6-11].

The term "teledentistry" was first used in 1997 to describe the profession of diagnosing patients remotely and offering treatment recommendations via video conferencing technology. Put differently, teledentistry refers to the application of information technology and telecommunications, similar to telehealth and telemedicine, for dental care, consultation, education, and public awareness. Additionally, teledentistry can help general dentists with specialty work and enhance services for underprivileged groups, such as those living in rural or less developed locations [12-14].

People's fear of infection prevented them from seeing dentists during the COVID-19 outbreak, and front-line healthcare providers, including dentists, experienced extreme unrest, worry, poor sleep, and a higher risk of mental illness. Social separation and house quarantine were advised by regulatory bodies as ways to limit close contact between people and the spread of sickness. Teledentistry was shown to be useful in reducing the risk of COVID-19 transmission, increasing healthcare efficiency, and triaging patients, even if it could not completely replace in-person dental operations. Its underutilization among dentists, however, emphasizes the necessity of education and knowledge sharing to boost dental professionals' interest and uptake [15-22].

Although the perceptions regarding the concept of teledentistry are relatively new and in the nascent stages of development in India, still dental clinicians and academicians are finding innovative and unique ways of practicing teledentistry. This article delves into the current state of awareness and adoption of teledentistry among dentists in Chennai, India. The study employs a comprehensive survey methodology, gathering insights from a diverse sample of dental practitioners within the region.

## Materials and methods

The institutional ethics committee (SRMIEC-ST0323-743) accepted the web-based descriptive cross-sectional study. A pre-validated computerized questionnaire (given in Appendices) was issued to dental practitioners in Chennai, India, during October 2023 and November 2023. Participants in the study comprised postgraduate dentistry students, general clinicians, specialists/consultants, and dental academicians from Chennai. The exclusion criteria included dentists who are not currently practicing or have retired.

The questionnaire was constructed using Google Forms (Google LLC, Mountain View, US). An e-invitation was distributed via social media (Facebook, WhatsApp, Instagram) to clarify the study's goal. Dentists in Chennai who consented to complete the questionnaire were added until the needed sample size was reached using a simple random sampling method.

The questionnaire was divided into two sections: the first collected professional and demographic information as well as communication preferences, and the second contained 26 five-point Likert-type questions divided into four categories: dental professionals' data security concerns, teledentistry and practice improvement, the usefulness of teledentistry for dental practice, and its usefulness for dental patients.

Sample size justification

According to a 2015 study by Mamatha et al., 27.3% of people were aware of teledentistry. By applying the formula below, the minimum sample size was estimated at 74. Considering the non-response rate, 90 was the final sample size for the study [5,6].

N = Z2 (1-α) p (1-p) / d2,

where n = sample size; z = level of confidence according to the standard normal distribution (for a level of confidence of 95%, z = 1.96; for a level of confidence of 99%, z = 2.575); p = estimated proportion of the population that presents the characteristic (when unknown, p = 0.5); d = tolerated margin of error (to know the real proportion within 5%). 

Statistical analysis

Data was entered into an Excel sheet (Microsoft Corp., Redmond, US) and subsequently analyzed using IBM SPSS Statistics for Windows, Version 21 (IBM Corp., Armonk, US). Chi-square tests were performed. Descriptive statistics, in terms of frequency (n) and percentage (%), were calculated to provide a comprehensive snapshot of the response distribution.

## Results

A total of 90 dentists in Chennai, India, responded to the electronic questionnaires, which were circulated between October 2023 and November 2023. The non-response rate was 20% and 15 dentists did not respond to the survey. The questionnaire was previously validated [23].

Demographic and work characteristics of respondents

The age group of 21 to 30 years accounted for about 45% of the respondents. Around 37 (41%) respondents had fewer than five years of professional experience, and more than half of them were general dentistry practitioners. Over 55 (61%) respondents were male. About 30 (33%) practitioners were employed in proper Chennai city, with the remaining practitioners working in smaller locations. Over 35% of the participants worked for more than eight hours per day. Nearly 73 (81.1%) participants worked in private practices, 10 (11.1%) in both public and private practices, and 12 (13.3%) in only academic practices.

Telemedicine and the use of the internet

Over 40 (44%) practitioners used the internet for about two to four hours for general purposes, out of which only 28 (31%) practitioners used it for dental/medical purposes during that same period of time. Of all the participants in this web-based questionnaire study, more than half of the dentists knew about teledentistry, and around 32 (35.6%) dentists practiced teledentistry.

Preferred methods of communication 

**Figure 1:**
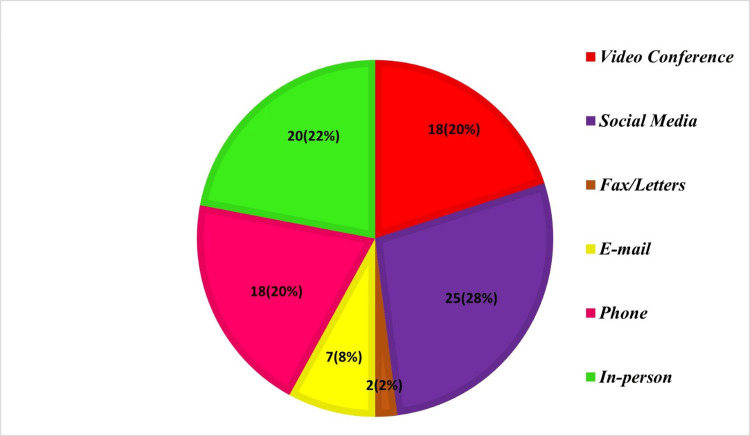
Preferred methods of communication

Since the development of teletechnology, there has been a significant shift in the forms of desired communication. Fax and letters are two examples of old-fashioned communication methods that now seem quite outmoded or non-preferred. In-person contact 20 (22%), phone 18 (20%), social media 25 (28%), and video conferencing 18 (20%) were the most common modes of communication selected by the participants. Email and fax were the least popular (or practically non-preferred) methods of communication among the practitioners (Figure 1). 

Practitioner’s perception of the capability of teledentistry to improve practice

A little more than half of the practitioners said that, in a clinical setting, teledentistry would be beneficial for correct diagnosis. Nearly 18 to 31 (23.7%-31%) doctors thought that patient referrals would be more effectively handled by teledentistry. The practitioners' opinions on teledentistry's capabilities were generally favorable. Merely six (7.9%) dentists expressed skepticism about it.

Practitioners’ perception of the usefulness of teledentistry for dental practice

The overwhelming majority of participants concluded that compared to a traditional referral system, teledentistry would save more time and improve clinical training and continuing education, which would be advantageous for dental practices. While over 15 to 28 (18%-35%) respondents thought that teledentistry would result in significant cost savings for dental practices, more time would be needed to schedule special visits for dental photos. On the other hand, nearly half of the participants were either not convinced or very little convinced by the idea of spending more time in treatment with the patient through teledentistry. There was disagreement over whether the infrastructure needed for teledentistry would be too expensive to set up.

Practitioners’ perception of the usefulness of teledentistry for patients

Most dental professionals agreed unanimously that teledentistry is good for patients. On the contrary, nearly 70% of the respondents said that patients living in rural or isolated areas would particularly benefit from teledentistry. Around 50% of the practitioners also felt that health insurance companies ought to fund teledentistry, and they affirmed that there are a few other benefits for patients from teledentistry, such as patient education and fewer in-office visits. The remaining questions evaluated the advantages of teledentistry for patients, and the results were generally positive. More than half of the participants thought that teledentistry would be convenient for patients and improve communication with them. It would also be helpful in monitoring their condition.

Concern of practitioners

**Figure 2:**
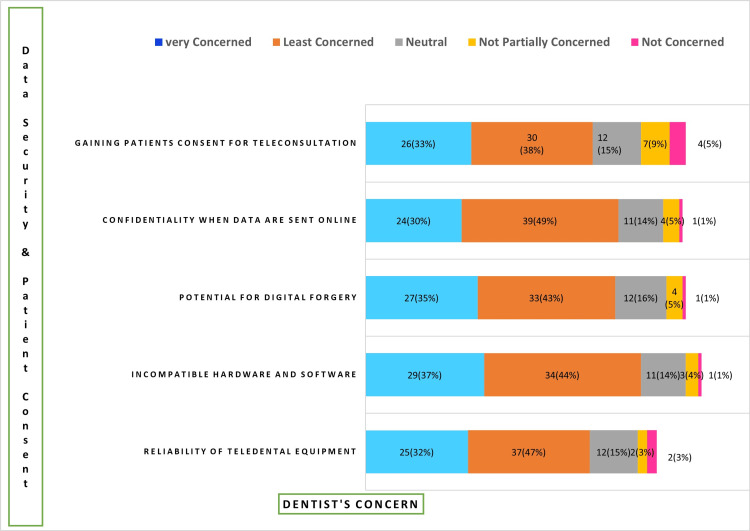
Concern of practitioners for data security and patient consent

Potential forgery of digital data becomes a greater risk when data is delivered online than issues with patient confidentiality, getting consent, incompatibility between software and hardware, or equipment dependability. This was also demonstrated by the study's findings, which showed that more than 27 (35%) participants expressed anxiety about digital forgeries. Concerns around patient confidentiality came next, with roughly 24 (30%) respondents expressing serious worries. Subsequently, around 25 (32%) participants voiced serious worries about the reliability of teledental equipment and 29 (37%) participants were concerned about the incompatibility of software and hardware. On the contrary, around four (5%) clinicians were not concerned about the confidentiality of the data sent online (Figure 2). 

Preferred dental specialty where teledentistry can be applied

**Figure 3:**
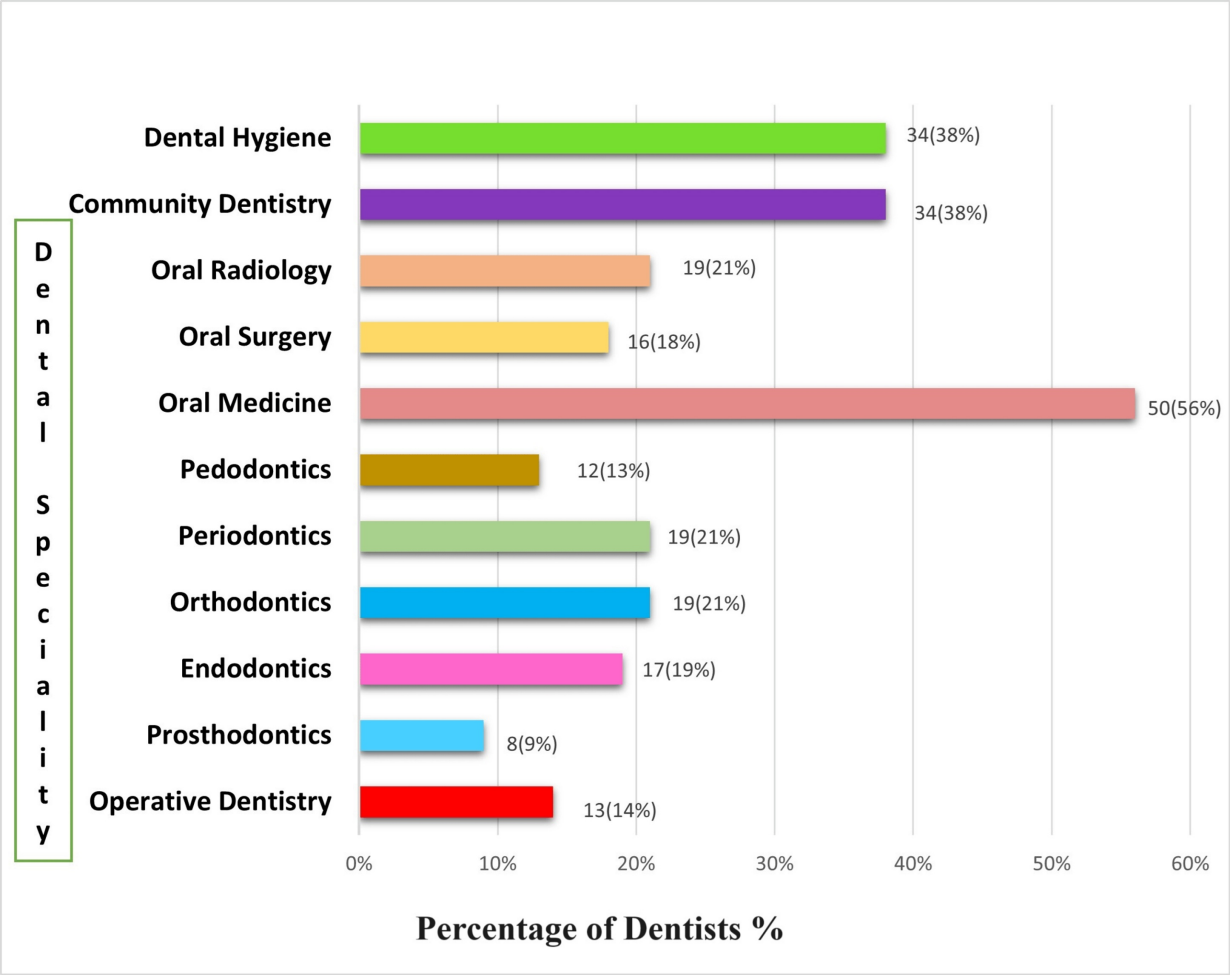
Preferred dental specialty where teledentistry can be applied

The survey's last question asked practitioners which dental specialty they believed would be the best candidates for teledentistry. More than 50 (56%) respondents ranked Oral Medicine and 34 (38%) ranked Dental Hygiene and Community Dentistry as their top priorities. Prosthodontics turned out to be the least favorable specialty, with eight (9%), to be dealt with teledentistry (Figure 3).

## Discussion

This study is most likely the first of its kind to look into how dental healthcare providers in Chennai perceive and are aware of telemedicine services. Dental care is given to patients via teledentistry as an alternative to in-person interactions [4]. The healthcare services industry has seen significant changes as a result of advancements in information and technology. There are various kinds of dentistry that use telemedicine, including patient dentistry, specialist dentistry, dentist data storage banks, student dentistry, dentist research centers, and several online methods like video conferencing, teleconferences, e-data, etc. [5,14,24].

Teledentistry, the intersection of dental care and telecommunication technologies, has emerged as a transformative force in the field of dentistry. As the world becomes increasingly interconnected, the dental profession is witnessing a paradigm shift in communication methods, clinical practices, and patient care. This discussion delves into a comprehensive analysis of a web-based questionnaire study that explores the perspectives of dental practitioners on teledentistry [25,26]. The study encompasses demographic data, practitioner awareness, utilization rates, perceived benefits, communication preferences, and concerns related to patient confidentiality and technology reliability.

The surveyed dental practitioners, predominantly within the age group of 21 to 30 years (45%), represent a significant proportion of the dental workforce. A noteworthy finding is that more than half of the respondents were general dentistry practitioners, with 37 (41%) having fewer than five years of professional experience. The majority of the participants, 55 (61%), were male, and a substantial portion of practitioners, 32 (35.6%), worked for over eight hours per day. This demographic breakdown offers a snapshot of the diverse group contributing to the study's insights.

A multifaceted analysis of internet usage patterns among practitioners reveals that while a substantial number spend two to four hours on the internet for general purposes, only 28 (31%) utilize it for dental/medical purposes within the same timeframe. Merely one (1%) relies exclusively on the internet for dental/medical consultations. This data underscores a potential gap in leveraging online resources for professional purposes within the dental community.

The study reveals that more than half of the surveyed dentists were aware of teledentistry and approximately 32 (35.6%) actively practiced it. This awareness gap and the relatively low adoption rate signal an opportunity for increased education and training to promote the benefits and integration of teledentistry into everyday dental practices [13,25].

As teletechnology reshapes communication methods, the study highlights a shift away from traditional forms such as faxes and letters. In-person contact, phone calls, social media, and video conferencing emerge as the preferred modes of communication among practitioners, with email and fax being the least favored methods. This shift mirrors broader trends in digital communication preferences across various industries.

A nuanced understanding of practitioner opinions on teledentistry unveils positive sentiments. More than half of the respondents believe that teledentistry would be beneficial for correct diagnosis in a clinical setting. About 18 to 31 (23.7%-40.8%) also express confidence in its ability to handle patient referrals more effectively. The positive outlook extends to perceived time savings, improved clinical training, and continuing education, suggesting that teledentistry could enhance overall dental practice efficiency [25,27].

However, a notable divergence emerges when considering spending more time in treatment with patients through teledentistry. Approximately half of the participants exhibit skepticism or reluctance raising questions about the perceived efficacy and practicality of this aspect of teledental care [27].

Practitioners' perceptions regarding the economic impact of teledentistry present a mixed picture. While over half anticipate significant cost savings for dental practices, concerns arise about the need for additional time to schedule special visits for dental photos. Disagreement persists on whether the infrastructure required for teledentistry is prohibitively expensive, posing a potential barrier to widespread adoption [20,21].

Despite varying opinions among practitioners, a consensus emerges on the benefits of teledentistry for patients. The majority believes that teledentistry is good for patients, particularly those in rural or isolated areas. This aligns with the perception that health insurance companies should fund teledentistry, highlighting a potential avenue for financial support [3,7,10].

Practitioners also recognize additional patient benefits, including enhanced education, reduced in-office visits, and improved communication. These positive perceptions underscore the potential of teledentistry to democratize access to dental care and improve overall patient experiences [28,29].

The study delves into practitioners' concerns related to teledentistry, revealing a hierarchy of worries. The foremost concern is the potential forgery of digital data, with 27 (35%) participants expressing anxiety about this risk. Patient confidentiality follows closely, with approximately 24 (30%) respondents voicing serious worries. Concerns about the reliability of teledental equipment and incompatibility between software and hardware rank third, with around 25 (32%) and 29 (37%), respectively, expressing significant apprehension [18,30,31].

A notable finding is that a small number of clinicians, four (5%), exhibit minimal concerns about the confidentiality of data transmitted online. This divergence in viewpoints emphasizes the need for standardized security measures and guidelines to address varying levels of comfort with digital technologies.

We would like to recommend that continuous collaboration among practitioners, policymakers, and technology developers is required to fully realize the promise of teledentistry and ensure its good influence on both dental professionals and the varied patient community in Chennai.

This study has various drawbacks. The sample size of only Chennai-based dentists limits the findings' generalizability to other regions. The reliance on self-reported data may cause response bias. Furthermore, the cross-sectional design captures a single point in time and does not account for changes in consciousness over time. 

Limitations of the study

Geographic limitation: One significant disadvantage of this study is its focus on Chennai dentists, which may limit its application to larger contexts due to differences in technology access, healthcare policies, and telemedicine support across different regions globally and within India.

Selection bias: The study's web-based questionnaire may result in selection bias, prioritizing individuals with internet access and digital skills. This may exclude less tech-savvy dentists, thus exaggerating telemedicine awareness among surveyed Chennai dentists and restricting the study's representation.

Response bias: Self-reported questionnaires introduce response bias because participants may prioritize socially acceptable responses over true opinions, particularly in professional settings where admitting ignorance may be perceived poorly, potentially inflating stated telemedicine awareness levels.

Cultural and policy differences: Local legislation, cultural perspectives on technology and healthcare, and legal frameworks all influence healthcare practices, including the use of telehealth. Findings from Chennai dentists may not apply to places with different cultural perspectives or regulatory settings, influencing telemedicine adoption rates appropriately.

The study's limitations, including its geographic scope, potential biases in participant selection and responses, and variations in cultural, and policy contexts, need addressing. Future research should strive for sample diversity, broader geographic coverage, and bias-reducing methodologies to enhance the finding’s applicability.

## Conclusions

The study emphasizes the enormous effort required to promote awareness and knowledge of teledentistry among dental practitioners. Targeted advertisements are vital for informing dentists and the general public about teledentistry. Finally, the study takes a detailed look at teledentistry from the standpoint of dental practitioners. Although awareness and adoption rates are improving, there are still issues and doubts that must be addressed. The positive opinions on patient benefits, cost savings, and enhanced efficiency point to a bright future for teledentistry. However, tackling challenges such as data security, patient confidentiality, and technology reliability is critical to its wider adoption.

Chennai, as a vibrant urban center, has a dental workforce that is generally in sync with technological advances. The fact that more than half of the dentists polled are aware of teledentistry, with a sizable proportion actively practicing it, indicates a favorable trend. However, the report reveals potential for additional education and training, particularly given the low adoption rates.

## Appendices

Questionnaire

1. Age

a)     20-34 year

b)     35-44 year

c)     45-54 year

d)     55-64 year

e)     >65 year

2. Gender

a)     Male

b)     Female 

c)     Others

3. Qualification

a)     Consultant/Specialist

b)     General dental practitioner

c)     Resident or graduate research

d)     Undergraduates

e)     Postgraduates

f)      Other

4. Work experience (in years)

a)     0-5 years

b)     6-10 years

c)     11-15 years

d)     >16 years

5. Location of the main job

a)     Major city

b)     City/town

c)     Remote area

6. Work setting of the main job

a)     Private

b)     Governmental

c)     Both (private & governmental)

d)     Academic

7. Working hours per week

a)     1-3 hour

b)     3-5 hour

c)     5-6 hour

d)     6-8 hour

8. Daily general-purpose use of the internet (in hours)

a)     <1 hour

b)     2-4 hour

c)     5-7 hour

d)     8-10 hour  

e)     >11 hour

9. Preferred communication tool in your dental practice

a)     Videoconference

b)     Social Media

c)     Fax/Letters

d)     Email

e)     Phone

f)      In person

10. Gaining patient consent for teleconsultation

a)     Very concerned

b)     Little concerned

c)     Not feeling either way

d)     Not particularly concerned

e)     Not concerned at all

11. Confidentiality when data are sent online

a)     Very concerned

b)     Little concerned

c)     Not feeling either way

d)     Not particularly concerned

e)     Not concerned at all

12. Potential for digital forgery

a)     Very concerned

b)     Little concerned

c)     Not feeling either way

d)     Not particularly concerned

e)     Not concerned at all

13. Incompatible hardware and software

a)     Very concerned

b)     Little concerned

c)     Not feeling either way

d)     Not particularly concerned

e)     Not concerned at all

14. Reliability of teledental equipment

a)     Very concerned

b)     Little concerned

c)     Not feeling either way

d)     Not particularly concerned

e)     Not concerned at all

15. Telemedicine would provide accurate diagnosis in a clinical setting

a)     Disagree strongly

b)     Disagree

c)     Neutral

d)     Agree

e)     Agree strongly

16. Telemedicine would help shorten the waiting list

a)     Disagree strongly

b)     Disagree

c)     Neutral

d)     Agree

e)     Agree strongly

17. Telemedicine would enhance guidelines and advice

a)     Disagree strongly

b)     Disagree

c)     Neutral

d)     Agree

e)     Agree strongly

18. Telemedicine would improve the interaction between peers

a)     Disagree strongly

b)     Disagree

c)     Neutral

d)     Agree

e)     Agree strongly

19. Telemedicine would provide a safe atmosphere for practicing dentistry (e.g., COVID-19 pandemic)

a)     Disagree strongly

b)     Disagree

c)     Neutral

d)     Agree

e)     Agree strongly

20. Telemedicine would make patient’s referral more efficient

a)     Disagree strongly

b)     Disagree

c)     Neutral

d)     Agree

e)     Agree strongly

21. Telemedicine would enhance clinical training and continuing education

a)     Disagree strongly

b)     Disagree

c)     Neutral

d)     Agree

e)     Agree strongly

22. Telemedicine would reduce costs for the dental practices

a)     Disagree strongly

b)     Disagree

c)     Neutral

d)     Agree

e)     Agree strongly

23. Telemedicine would increase treatment time spent with the patient

a)     Disagree strongly

b)     Disagree

c)     Neutral

d)     Agree

e)     Agree strongly

24. Telemedicine would necessitate an extra appointment for taking photographs

a)     Disagree strongly

b)     Disagree

c)     Neutral

d)     Agree

e)     Agree strongly

25. Telemedicine would save time compared with a referral letter

a)     Disagree strongly

b)     Disagree

c)     Neutral

d)     Agree

e)     Agree strongly

26. Telemedicine would be too expensive to set up

a)     Disagree strongly

b)     Disagree

c)     Neutral

d)     Agree

e)     Agree strongly

27. Telemedicine would provide adequate diagnostic information

a)     Disagree strongly

b)     Disagree

c)     Neutral

d)     Agree

e)     Agree strongly

28. Telemedicine would save money for patients

a)     Disagree strongly

b)     Disagree

c)     Neutral

d)     Agree

e)     Agree strongly

29. Telemedicine would improve communication with patients

a)     Disagree strongly

b)     Disagree

c)     Neutral

d)     Agree

e)     Agree strongly

30. Telemedicine would be helpful for patient education

a)     Disagree strongly

b)     Disagree

c)     Neutral

d)     Agree

e)     Agree strongly

31. Telemedicine would help to avoid unnecessary travel to the dental clinic

a)     Disagree strongly

b)     Disagree

c)     Neutral

d)     Agree

e)     Agree strongly

32. Telemedicine would be helpful in monitoring the patient's condition

a)     Disagree strongly

b)     Disagree

c)     Neutral

d)     Agree

e)     Agree strongly

33. Telemedicine would be useful for patients in remote areas

a)     Disagree strongly

b)     Disagree

c)     Neutral

d)     Agree

e)     Agree strongly

34. Telemedicine should be covered by dental insurance plans.

a)     Disagree strongly

b)     Disagree

c)     Neutral

d)     Agree

e)     Agree strongly

35. Telemedicine can be applied in which branch of dentistry? (choose one or more options)

a)     Operative dentistry

b)     Prosthodontics

c)     Endodontics

d)     Orthodontics

e)     Periodontics

f)      Pedodontics

g)     Oral medicine

h)     Oral surgery

i)      Oral radiology

j)      Community dentistry

k)     Dental hygiene

## References

[1] Alhudaithi AS, Alsughier Z, Alzaidan H, Aldhelai TA (2023). Children's oral health status among urban and rural areas of Qassim region, Saudi Arabia: a cross-sectional study. Cureus.

[2] Chen HF, Lee HE, Chen IT, Huang YT, Ho PS, Karim SA (2023). Rural-urban disparities in the incidence and treatment intensity of periodontal disease among patients with diabetes. Front Public Health.

[3] Dasgupta A, Deb S (2008). Telemedicine: a new horizon in public health in India. Indian J Community Med.

[4] Malpe M, Choudhari SG, Nagtode N, Muntode Gharde P (2024). Beyond the chair: exploring the boundaries of teledentistry. Cureus.

[5] Boringi M, Waghray S, Lavanya R (2015). Knowledge and awareness of teledentistry among dental professionals - a cross sectional study. J Clin Diagn Res.

[6] Brunello G, Gurzawska-Comis K, Becker K, Becker J, Sivolella S, Schwarz F, Klinge B (2021). Dental care during COVID-19 pandemic: follow-up survey of experts' opinion. Clin Oral Implants Res.

[7] Blum IR (2021). Urgent dental care and oral health under the clouds of COVID-19. Prim Dent J.

[8] Kumar G, Rehman F, Al-Muzian L, Farsi D, Hiremath S (2021). Global scenario of teledentistry during COVID-19 pandemic: an insight. Int J Clin Pediatr Dent.

[9] da Silva HE, Santos GN, Leite AF (2021). The role of teledentistry in oral cancer patients during the COVID-19 pandemic: an integrative literature review. Support Care Cancer.

[10] Hariyani N, Shanbhag N, Wijayati EW, Prananta AW, Setyowati D, Palupi R (2022). Teledentistry and online referral system in Indonesian primary health care center during the COVID-19 pandemic: a narrative review. J Int Soc Prev Community Dent.

[11] Lin GS, Koh SH, Ter KZ, Lim CW, Sultana S, Tan WW (2022). Awareness, knowledge, attitude, and practice of teledentistry among dental practitioners during COVID-19: a systematic review and meta-analysis. Medicina (Kaunas).

[12] Jampani ND, Nutalapati R, Dontula BS, Boyapati R (2011). Applications of teledentistry: a literature review and update. J Int Soc Prev Community Dent.

[13] Aboalshamat KT (2020). Awareness of, beliefs about, practices of, and barriers to teledentistry among dental students and the implications for Saudi Arabia vision 2030 and coronavirus pandemic. J Int Soc Prev Community Dent.

[14] Khokhar RA, Ismail WA, Sunny A (2022). Awareness regarding teledentistry among dental professionals in Malaysia. Biomed Res Int.

[15] Mahdavi A, Atlasi R, Naemi R (2022). Teledentistry during COVID-19 pandemic: scientometric and content analysis approach. BMC Health Serv Res.

[16] Abbas B, Wajahat M, Saleem Z, Imran E, Sajjad M, Khurshid Z (2020). Role of teledentistry in COVID-19 pandemic: a nationwide comparative analysis among dental professionals. Eur J Dent.

[17] Daniel SJ, Kumar S (2014). Teledentistry: a key component in access to care. J Evid Based Dent Pract.

[18] Haider MM, Allana A, Allana RR (2020). Barriers to optimizing teledentistry during COVID-19 pandemic. Asia Pac J Public Health.

[19] Sycinska-Dziarnowska M, Maglitto M, Woźniak K, Spagnuolo G (2021). Oral health and teledentistry interest during the COVID-19 pandemic. J Clin Med.

[20] Wakhloo T, Reddy GS, Chug A, Dhar M (2020). Relevance of teledentistry during the COVID-19 pandemic. J Family Med Prim Care.

[21] Santana LA, Santos MA, Albuquerque HI, Costa SF, Rezende-Silva E, Gercina AC, Takeshita WM (2020). Teledentistry in Brazil: a viable alternative during COVID-19 pandemic. Rev Bras Epidemiol.

[22] Ghai S (2020). Teledentistry during COVID-19 pandemic. Diabetes Metab Syndr.

[23] Soegyanto AI, Wimardhani YS, Maharani DA, Tennant M (2022). Indonesian dentists' perception of the use of teledentistry. Int Dent J.

[24] Cheuk R, Adeniyi A, Farmer J, Singhal S, Jessani A (2023). Teledentistry use during the COVID-19 pandemic: perceptions and practices of Ontario dentists. BMC Oral Health.

[25] Howell SE, Fukuoka B (2022). Teledentistry for patient-centered screening and assessment. Dent Clin North Am.

[26] Hung M, Lipsky MS, Phuatrakoon TN, Nguyen M, Licari FW, Unni EJ (2022). Teledentistry implementation during the COVID-19 pandemic: scoping review. Interact J Med Res.

[27] Islam MR, Islam R, Ferdous S, Watanabe C, Yamauti M, Alam MK, Sano H (2022). Teledentistry as an effective tool for the communication improvement between dentists and patients: an overview. Healthcare (Basel).

[28] Fornaini C, Rocca JP (2022). Relevance of teledentistry: brief report and future perspectives. Front Dent.

[29] Wadia R (2021). Teledentistry. Br Dent J.

[30] Saraswati S, Bhowmick D, Upasana K, Pravin KS, Srivastava S, Smita Smita (2022). A study to assess patients' perception and acceptance of teledentistry for care during the COVID-19 pandemic. J Pharm Bioallied Sci.

[31] Shigekawa E, Fix M, Corbett G, Roby DH, Coffman J (2018). The current state of telehealth evidence: a rapid review. Health Aff (Millwood).

